# The role of the complement system in primary membranous nephropathy: A narrative review in the era of new therapeutic targets

**DOI:** 10.3389/fimmu.2022.1009864

**Published:** 2022-10-24

**Authors:** Benjamin Y. F. So, Gary C. W. Chan, Desmond Y. H. Yap, Tak Mao Chan

**Affiliations:** Division of Nephrology, Department of Medicine, Queen Mary Hospital, The University of Hong Kong, Hong Kong, Hong Kong, SAR, China

**Keywords:** complement, primary membranous nephropathy, glomerulonephritis, nephrotic syndrome, chronic kidney disease, mannose-binding lectin pathway, alternative pathway, classical pathway

## Abstract

Primary membranous nephropathy (MN) is an important cause of nephrotic syndrome and chronic kidney disease (CKD) in the adult population. Although the discovery of different autoantibodies against glomerular/podocytic antigens have highlighted the role of B cells in the pathogenesis of MN, suboptimal response or even resistance to B cell-directed therapies occurs, suggesting that other pathophysiological mechanisms are involved in mediating podocyte injury. The complement system plays an important role in the innate immune response to infection, and dysregulation of the complement system has been observed in various kidney diseases. There is compelling evidence of complement cascade activation in primary MN, with the mannose-binding lectin (MBL) and alternative pathways particularly implicated. With appropriate validation, assays of complements and associated activation products could hold promise as adjunctive tools for non-invasive disease monitoring and prognostication. While there is growing interest to target the complement system in MN, there is concern regarding the risk of infection due to encapsulated organisms and high treatment costs, highlighting the need for clinical trials to identify patients most likely to benefit from complement-directed therapies.

## Introduction

Membranous nephropathy (MN) is the most common cause of nephrotic syndrome in the adult population worldwide. Up to 12 per million adults are affected each year, although there is significant regional variation (1). Clinically, MN presents with proteinuria and varying degrees of renal impairment, and some present with a full-blown nephrotic syndrome with proteinuria, hypoalbuminaemia, peripheral oedema and hyperlipidaemia. A subset of patients develop chronic kidney disease (CKD) which could progress to end-stage kidney disease (ESKD) (2).

MN demonstrates characteristic histopathological features on kidney biopsy. On light microscopy, glomerular basement membrane thickening is demonstrated, together with the presence of epimembranous spikes on silver stain. Immunofluorescence typically reveals granular staining for IgG along the capillary loop; these are typically IgG4 deposits in primary MN, although IgG1 and IgG3 may also be present in earlier stages of primary MN. Salient to this review, C3 deposition is also commonly seen on immunofluorescence. Electron microscopy confirms the diagnosis by the presence of subepithelial electron-dense deposits together with other features such as diffuse effacement of podocyte foot processes (3).

Classically, MN diagnosed by histology is categorized into either primary or secondary forms. The latter refers to cases of MN that are secondary to another systemic disease, such as an underlying malignancy, an autoimmune disease (such as systemic lupus erythematosus), a chronic infection (such as hepatitis B or C), or drugs (such as non-steroidal anti-inflammatory drugs). Accordingly, the term “primary MN” was used to describe cases that were thought to be idiopathic (2). Recent research suggests that this distinction may be overly simplistic and arbitrary. Indeed, both primary and secondary MN have been shown, both by animal models and in human subjects, to arise from antibodies directed against glomerular antigens, with development of immune deposits, and downstream pathogenic mechanisms resulting in podocyte injury and the clinical manifestations (4). Among the previously “idiopathic” cases, up to 60-70% of cases were found to be the result of antibodies directed against the glomerular phospholipase A2 receptor (PLA2R) antigen (5). In a smaller proportion of patients, thrombospondin type 1 domain containing 7A (THSD7A), semaphorin 3b (Sema3b), protocadherin 7 or neural epidermal growth factor-like 1 (NELL1) (6–9) have been identified as the target antigen. Circulating antibodies against the aforementioned antigens have been identified in the sera of patients with MN. Meanwhile, in some secondary causes of MN, exostosin 1 and exostosin 2 (EXT1 and EXT2) may also serve as target antigens, although specific antibodies directed against EXT1 and EXT2 have yet to be isolated from patient sera (10).

The identification of target glomerular antigens in primary MN has revolutionized disease diagnosis, classification, prognostication and treatment. MN is now conceptualized as a predominantly B cell disorder with disease-causing antibodies. Some of these antibodies, such as anti-PLA2R antibodies, are directly correlated with the clinical disease course. Anti-PLA2R antibodies have been used as a “liquid biopsy” for patients presenting with proteinuria, obviating the need for kidney biopsy in select patients; anti-PLA2R antibodies may also be used to track the clinical course and detect relapses at an earlier time (11–13). Accordingly, B cell-directed therapies, especially B cell depleting agents like rituximab, have become popular treatment options for primary MN with proven efficacy.

Disease-specific antibodies lead to the clinical manifestations of primary MN through a variety of downstream pathways, of which the complement system has been the best characterized. A growing body of research is re-evaluating the role of complement pathways in primary MN associated with particular glomerular antigens, in light of the plethora of new antigens being discovered that challenge the previous monolithic understanding of idiopathic primary MN. This narrative review will review the evidence regarding the role that complement pathway activation plays in the pathogenesis of primary MN, and highlight its potential role in disease diagnosis, prognostication, monitoring and treatment.

## The complement system and kidney disease

**Figure 1:**
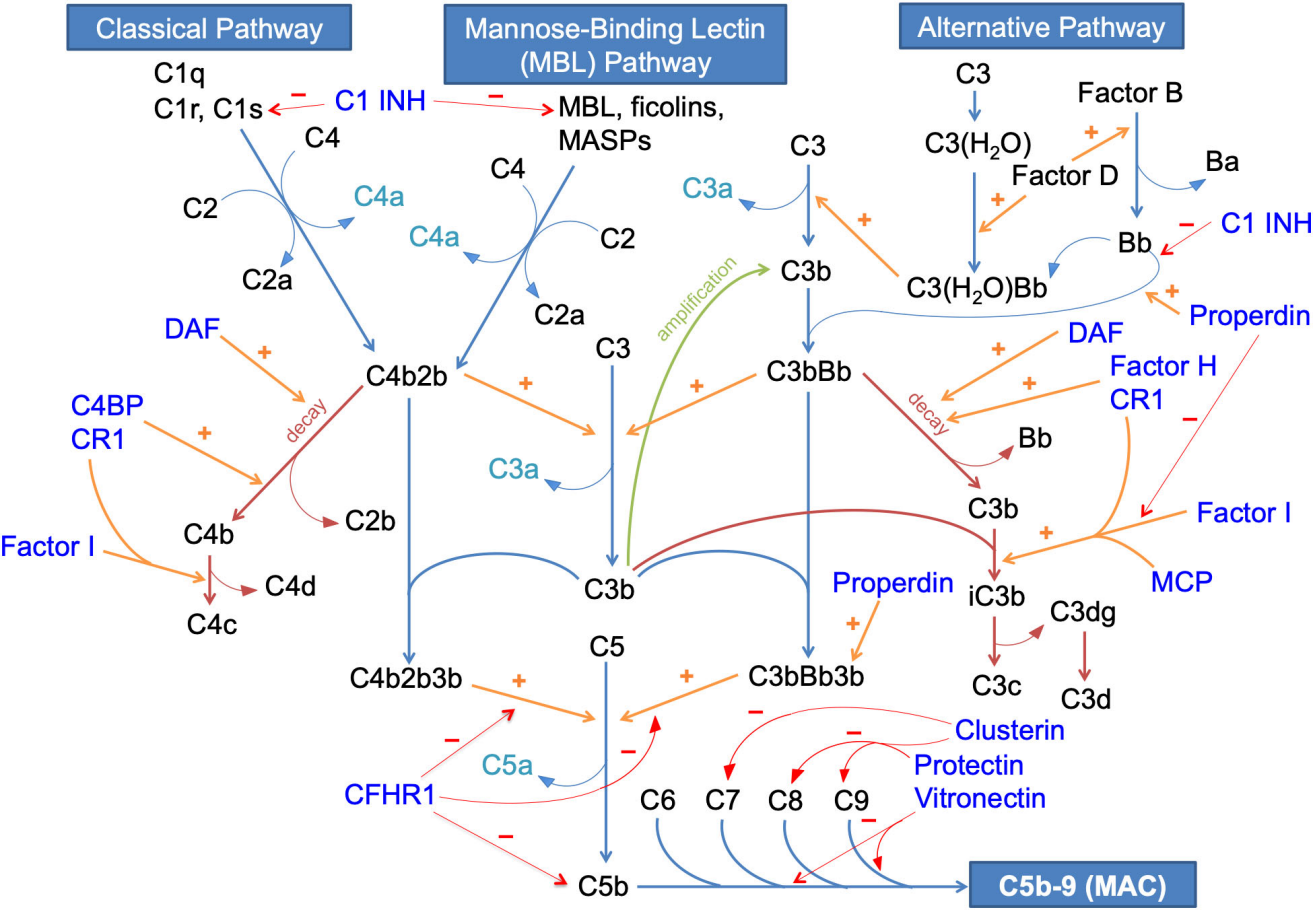
The complement system in humans. C1 INH, C1 inhibitor; C4BP, C4 binding protein; CFHR1, complement factor H-related protein 1; CR1, complement receptor 1; DAF: decay accelerating factor; MAC, membrane attack complex; MASPs, mannose-binding lectin-associated serine proteases; MBL, mannose-binding lectin; MCP, membrane cofactor protein; amplification pathways are shown in green; decay pathways are shown in maroon; inhibitory pathways are shown in red; stimulatory/catalytic pathways are shown in orange; complement regulatory proteins (CRPs) are shown in indigo; anaphylatoxins are shown in cyan; all other complements and related products are shown in black.

The complement system is a tightly regulated component of the innate immune system intended to enhance the abilities of antibodies and phagocytic cells to attack cell membranes of pathogens, clear microbial organisms and damaged cells, and promote inflammation. Three pathways, namely the classical pathway, alternative pathway and mannose-binding lectin (MBL) pathway, can activate the complement cascade. All three pathways converge on a common terminal pathway to result in the synthesis of a membrane attack complex (MAC), which can cause pores in pathogen cell membranes resulting in cell lysis (14, 15). The pathways that make up the complement cascade are shown in **Figure 1**.

The classical pathway of complement is triggered by the interaction between C1q, which is part of a C1qrs complex with 2 C1r molecules and 2 C1s molecules, and the Fc receptors of immunoglobulin molecules such as IgM or IgG. A conformational change of C1s allows for the cleavage of C4 into C4a and C4b, and C2 into C2a and C2b. The larger fragments of these 2 key cleavages combine to generate the C3 convertase C4b2b, which further cleaves C3. C3b generated from cleavage of C3 then binds to C3 convertase to form the C5 convertase (C4b2b3b) that cleaves C5. Among these cleaved complement activation products, C3a and C5a are anaphylatoxins that attract phagocytic cells to sites of inflammation, while C3b may also function as an opsonin. C5b interacts with other terminal pathway proteins, including C6, C7, C8 and C9 to form the C5b-9 complex, better known as the MAC (14, 15).

The MBL pathway of complement is analogous to the classical pathway, and is triggered by the interaction between MBL and carbohydrate motifs on bacteria. Like C1r and C1s, mannose-binding lectin serine proteases (MASPs) can cleave C4 and C2 to generate the C3 convertase C4b2b (14–16).

The alternative pathway of complement, in comparison, is constitutively active at a low level in normal humans, and can amplify rapidly in response to pathogens. The alternative pathway is triggered when C3b binds to a water molecule. Factor B and D are recruited, and factor D enzymatically cleaves factor B to form Bb, an active serine esterase that can cleave C3 into C3a and C3b. C3b binds with Bb to form C3bBb, the alternative pathway C3 convertase which can break down C3 further. This creates an amplification loop whereby more C3 products are formed that can further propagate the pathway. C3bBb forms a multimeric complex with C3b to form the C5 convertase C3bBbC3b, thereby cleaving C5 to generate the terminal complement pathway (14, 15).

To prevent the alternative pathway from spiraling out of control, several regulatory proteins and pathways are normally present to keep a check on alternative pathway activity. These include both regulatory proteins in the blood and those that are membrane-bound. Decay-accelerating factor (DAF), a membrane-bound regulator catalyzes the degradation of both classical pathway and alternative pathway-associated C3 convertases (C4b2b and C3bBb). Membrane cofactor protein (MCP) is a surface-expressed protein that serves as a cofactor for factor I, which irreversibly cleaves C3b to iC3b thus preventing formation of the alternative complement C3 convertase. It has an equivalent murine homologue in the form of Crry (14, 15). Factor H, a plasma protein, is the most important regulator of the alternative pathway in the fluid phase, and works by accelerating the decay of C3bBb convertase and again by catalyzing the inactivation of C3b to iC3b by factor I. It may also bind to polyanionic glycosaminoglycans on exposed surfaces such as the glomerular basement membrane to regulate the local alternative pathway of complement (17). Complement receptor 1 (CR1) is a surface-expressed regulatory protein with similar functions. Properdin is the only positive regulator of the alternative pathway, and functions by promoting binding of factor Bb to C3b to form the alternative pathway C3 convertase C3bBb, by inhibiting factor I-mediated inactivation of C3b, and by stabilizing the alternative pathway C5 convertase C3bBb3b (18).

The classical and MBL pathways are also subjected to regulation, although this is less important than alternative pathway regulation since these pathways require an antigenic trigger. The classical pathway C3 convertase C4b2b undergoes decay mediated by the membrane-bound DAF and CR1, and by C4 binding protein (C4BP) in the fluid phase (19). C1 inhibitor can regulate the classical and MBL pathways as it can irreversibly bind to and inactivate C1r, C1s, MASP1 and MASP2, and also the alternative pathway by preventing association of factor Bb with C3b, thus preventing formation of the alternative pathway C3 convertase (14–16, 20). The terminal pathway is also regulated at multiple levels. The complement factor-H related proteins (CFHRs) are a group of proteins, structurally related to factor H, that typically display only weak regulatory ability of complement pathways (21). However, though it has not been shown to regulate the alternative pathway, complement factor H-related protein 1 (CFHR1) is an inhibitor of the C5 convertases C4b2b3b and C3bBb3b, and also prevents membrane deposition of C5b to prevent downstream formation of MAC (22). Meanwhile, the proteins clusterin, protectin and vitronectin bind to various constituent components of MAC to prevent its formation (23–26).

The archetypal example of a kidney disease caused by complement dysregulation is C3 glomerulopathy. C3 glomerulopathy is a rare kidney disease characterized by dysregulated activation of the alternative pathway. This could be due to development of a C3 nephritic factor, an autoantibody that binds to the alternative pathway C3 convertase C3bBb to stabilize it against factor H-mediated decay acceleration. Mutations in the factor H gene or other components of the alternative pathway of complement could also result in complement pathway dysregulation (27). However, varying degrees of complement pathway activation have been shown in different glomerulopathies, including primary MN and IgA nephropathy, as well as glomerulonephritides secondary to underlying systemic diseases such as lupus nephritis and anti-neutrophil cytoplasmic antibody (ANCA)-associated vasculitis (28–30).

## Evidence of complement pathway activation in membranous nephropathy

### The role of the terminal complement pathway in proteinuria and kidney injury in membranous nephropathy

There is unequivocal evidence from animal and human cell line models, as well as patients with primary MN, implicating complement pathway activation in the pathogenesis of proteinuria and kidney injury. Bioinformatics analysis in MN has shown that differentially expressed genes in MN were preponderantly associated with complement cascade-related immune responses (31). Regardless of the initial pathway of complement activation, it is clear that the final, common terminal complement pathway inflicts damage to kidney cells, especially in the glomeruli, resulting in the clinical manifestations of proteinuria and renal impairment.

C3 deposits, concurrent with pathognomonic IgG deposits, are commonly seen in primary MN, and seem to be correlated with increased proteinuria (32). C3, the first component of the final common pathway of complement, is the most abundantly expressed complement protein in the glomeruli of patients with MN, with high spectral counts exceedingly even those of target glomerular antigens such as PLA2R (33). In the passive model of Heymann nephritis, a commonly employed murine model of MN, pretreatment with cobra venom factor to maintain undetectable circulating C3 levels prior to induction of nephritis with injection of sheep antibody resulted in development of subepithelial immune deposits without C3, but without manifestation of any degree of proteinuria (34). Complement blockade resulted in preserved podocyte slit diaphragm morphology on electron microscopy, and preservation of nephrin and actin-associated nephrin on immunofluorescence (35). The findings from Heymann nephritis are corroborated by human cell lines, as the *in vitro* cytotoxicity of anti-PLA2R antibodies derived from donor sera on human kidney cells was greatly enhanced by the addition of rabbit complement, highlighting the pathogenic role of complement pathways (36). Furthermore, C3 deposition was correlated with increased rate of seropositivity for anti-PLA2R antibodies, and urinary levels of C5a were correlated with anti-PLA2R antibody titres and the level of proteinuria in seropositive patients, confirming a link between the pathogenic antibodies and subsequent complement-mediated tissue injury (32, 37). The C3a and C5a anaphylatoxins have been shown to be important effectors of complement-mediated damage in primary MN. Both C3a and C5a are elevated in the plasma of patients with MN (38). Overexpression of the C3a receptor (C3aR) has been demonstrated in human glomeruli exposed to plasma from patients with primary MN, and this was correlated with proteinuria, elevated serum creatinine and non-response to treatments. In rats with Heymann nephritis, blockade of the C3aR by C3aR antagonists led to attenuation of proteinuria, reduced deposition of electron-dense deposits, and improvements in foot process effacement and glomerular basement membrane thickening (39). Compared to patients with nephrotic syndrome who were diagnosed with focal segmental glomerulosclerosis (FSGS) or minimal change disease (MCD) on renal biopsy, patients with primary MN had several fold higher levels of serum C3a and C5a, illustrating that complement activation is a defining feature of MN that distinguishes it from other immune-mediated glomerulonephritides (40).

The formation of MAC appears central to the pathogenesis of glomerular damage and proteinuria. Laser microdissection of glomeruli obtained from human renal biopsy specimens show increased deposition of MAC in the glomeruli of patients with MN (41). In rat models of MN, including in Heymann nephritis, MAC deposition was associated with development of heavy proteinuria. Proteinuria persisted despite cessation of MAC assembly by pharmacologic means, suggesting that once MAC is formed, it persists in the glomerulus and continues to cause proteinuria for a protracted period of time (42–44). Induction of subepithelial deposits in C5-deficient mice resulted in reduced degrees of albuminuria, though it was not completely abolished (45). Development of proteinuria in the Heymann nephritis model was prevented with C6 depletion with a monoclonal antibody against murine C6 that prevented formation of MAC (46). The glomerular deposition of MAC is likewise mirrored by an increase in urinary excretion of MAC, which is consistently observed in the Heymann nephritis model (47).

Nucleated cells in the glomeruli are more resilient compared to erythrocytes, and multiple MAC lesions are required in order to cause direct cellular lysis (48). Sublytic damage inflicted by MAC may occur through alteration of intracellular calcium concentrations in glomerular visceral epithelial cells, changing their function and morphology. This manifests as effacement of foot processes with villous transformation, thus altering the permselectivity of the glomerular filtration barrier (42, 43, 49). Additionally, MAC activates phospholipases and stimulates the release of arachidonic acid and synthesis of glomerular eicosanoids including prostaglandins and thromboxanes (50). Thromboxane A2 has previously been shown in models of MN to increase glomerular transcapillary pressure giving rise to increased protein excretion (51). Sublytic injury due to MAC in the glomeruli may be modified by other factors. For example, cytochrome P450 2B1 may mediate complement-mediated sublytic injury through increasing reactive oxygen species, cytotoxicity and collapse of the actin cytoskeleton. In a rat model of MN, administration of the cytochrome P450 inhibitor cimetidine reduced development of reactive oxygen species and protected against development of proteinuria (52). In addition, complement stimulation of podocytes in kidney tissue obtained from patients with MN, as well as in a Heymann nephritis model, led to the development of cellular pyroptosis, a form of pro-inflammatory programmed cell death mediated by the innate immunity, as evidenced by increased mRNA levels of pyroptosis-associated genes (53).

Complement-mediated kidney injury is not limited to the glomeruli, but may extend to other compartments. Studies have shown that C3 can be formed in the renal tubulointerstitium *in situ*, often in response to local inflammation (37, 54). This is particularly marked in diseases with generalized renal inflammation, such as in lupus nephritis. Intraglomeular synthesis of C3 has been identified in the Heymann nephritis model (55). Likewise, in human primary MN, C3 synthesis occurs in proximal tubular cells, particularly in patients with more advanced renal failure. Although renal C3 synthesis in MN was not correlated with the degree of proteinuria *per se*, it was proportional to the degree of urinary MAC excretion, suggesting that *in situ* C3 synthesis might promote further expression of MAC in the tubules leading to further tubulointerstitial damage (56). The positive complement regulator properdin may play a pivotal role in complement activation and MAC deposition in the proximal tubular epithelial cells (57). The MAC can then stimulate collagen gene expression in renal tubular epithelial cells, leading to progressive tubulointerstitial fibrosis (58).

### The role of the classical complement pathway in membranous nephropathy

In humans, primary MN is mediated primarily *via* IgG4 immunoglobulins, including the anti-PLA2R antibody. Unlike other IgG isoforms, IgG4 cannot directly activate the classical pathway. Thus, it is generally presumed that the classical pathway is not involved in the pathogenesis of primary MN. Recent studies show that in a small number of patients, the presence of anti-PLA2R IgG1, IgG2 and IgG3 antibodies may be associated with increased levels of complement-mediated cytotoxicity *in vitro* (36). Although these IgG forms could theoretically activate the classical pathway, the role of the classical pathway in PLA2R-associated disease remains unproven. Meanwhile, the antibodies reactive to EXT1 and EXT2 antigens, which have been associated with cases of secondary MN, are IgG1 and may therefore activate the classical complement pathway more readily (33). Interestingly, studies of the rare segmental variant of primary MN, characterized by only segmental subepithelial immune deposits, showed presence of only IgG1 and IgG3 immunoglobulin deposits together with C1q positivity, suggesting activation of the classical complement pathway (59). One study that included over two hundred patients with primary MN reported that patients with C1q deposits seen on immunofluorescence had lower systemic IgG levels and higher IgA, IgM and C3c, but they did not differ from C1q-negative comparators in terms of renal function or proteinuria, suggesting that involvement of the classical pathway does not seem to have a major impact on clinical manifestations (60). It is increasingly clear that the immunopathogenesis of MN, be it primary or secondary, is variable depending on the target glomerular antigen involved.

### Mannose-binding lectin and alternative pathway activation in membranous nephropathy

Since the classical pathway is not usually activated in most cases of primary MN, it follows that complement activity must occur *via* activation of the MBL pathway or through dysregulation of the alternative pathway. Consistent with this hypothesis, in a study of kidney biopsy specimens of patients with MN, all patients had positive staining for C3, C4 and at least one component of the MBL pathway. All of them showed negative or trace staining for C1q, implying that the classical pathway did not play a major role (41).

MBL and hypogalactosylated IgG have been shown to be important components of the subepithelial deposits in primary MN (15). In a cell culture model of MN, anti-PLA2R antibody positive sera induced proteolysis of the podocyte proteins synaptopodin and NEPH1, through activation of the MBL pathway with MAC formation, in a glycosylation-dependent manner; galactose-deficient IgG4 titres were correlated with total anti-PLA2R antibody levels and proteinuria (61). Serum MBL levels and C4d were also correlated with increased proteinuria only in PLA2R-seropositive patients, but urinary levels of MBL and C4d were associated with proteinuria in both seropositive and seronegative patients (62). Inoculation of human anti-THSD7A antibodies activate the MBL pathway in mice, as evidenced by increased levels of complement pathway end-products together with MBL, and induced lesions of MN in murine kidneys. On immune staining, MBL, C3b, and C5b-9 staining was markedly increased in these mice, showing the pathogenic role of the MBL pathway (63). The prevalence and staining intensity of MBL deposits were also higher in patients who had positive glomerular staining for disease-causing antigens such as PLA2R and THSD7A, and MBL deposits were associated with unfavorable outcomes (64). Serum/plasma levels of MBL, MASP-1 and MASP-2 were higher in patients with positive anti-PLA2R antibodies compared to seronegative patients. Complete remission as defined by proteinuria endpoints was more likely to occur in seropositive patients (treated with various combinations of corticosteroids, cyclophosphamide, cyclosporine and/or tacrolimus) who had lower levels of MBL, MASP-1 and MASP2 at baseline (65). Polymorphisms in the *MBL2* gene were more commonly observed in patients with primary MN in one study, although correlations between *MBL2* genotype, circulating MBL levels, anti-PLA2R seropositivity, and proteinuria were not made in this study (66).

Since there are case reports of MN developing in patients with complete MBL deficiency, due to sequence polymorphisms in the promoter and the coding region, the alternative pathway must be involved in at least some cases of MN (67). The alternative pathway of complement is constitutively active through low-grade hydrolysis of C3. Pathophysiologically, dysfunction of the alternative pathway usually occurs due to congenital or acquired deficiencies of complement regulatory proteins, or resistance to their effects such as due to the development of autoantibodies. For example, in a murine model of MN induced by immunization with the noncollagenous domain of the α-3 chain of collagen, mice that were deficient in factor B and thus lacking a functional alternative pathway did not develop albuminuria or deposits of MAC, despite the formation of similar subepithelial IgG deposits (45). In human patients with THSD7A-associated MN, C3b fixation by THSD7A subepithelial immune complexes was completely abolished in factor B-depleted sera (68). Alterations in factor B have further been reported in both anti-PLA2R antibody-associated primary MN and in seronegative cases. Circulating levels of factor Ba, the breakdown product of factor B, as well as the total levels of factor B, were higher in patients with MN who were seropositive for anti-PLA2R antibody, compared to seronegative patients. Lower levels of factor Ba and factor Bb were associated with increased rate of complete remission of proteinuria in seropositive patients, and the decrease in factor B levels with time was linearly correlated with the fall in proteinuria (65). In other studies, serum complement factor Bb titres and urine properdin levels were associated with proteinuria only in patients with MN who were seronegative for anti-PLA2R antibody, but not in seropositive patients (38). Part of the dysfunction of factor B in MN could be antibody-mediated. For example, the alternative pathway was found to be more active in MN patients with anti-beta-2-glycoprotein I antibody (69). This is corroborated by findings that, in patients with the antiphospholipid syndrome, low complement levels are common; and in patients with lupus nephritis, the serum level of factor Bb and anti-beta-2-glycoprotein I antibody titres were correlated (70).

Factor H is an important regulator of the alternative pathway that is often implicated in kidney diseases, as it is solely responsible for regulating alternative pathway activation on surfaces that do not harbor any complement regulators, including the exposed surfaces of the glomerular basement membrane. Urinary factor H levels were elevated in MN compared with healthy controls (71). Laser microdissection and mass spectrometry of glomerular antigens in primary MN showed high spectral counts of factor H, complement factor H-related proteins 1 and 5 (CFHR1 and CFHR5), properdin and vitronectin in PLA2R-associated disease, and these were more abundant in PLA2R-associated MN than in exostosin 1 or 2 mediated MN (33). Heparan sulphate chains in the glomerular basement membrane normally recruit plasma factor H to regulate the local alternative pathway of complement. Expression of heparanases leading to loss of glomerular heparan sulphate has been associated with complement deposition and proteinuria in Heymann nephritis (72). Several reports further suggested that anti-complement factor H (anti-CFH) antibodies may be expressed in MN, although the evidence is conflicting. An initial case series reported that anti-CFH antibodies were possibly tied with deterioration in renal function in spite of anti-PLA2R antibody seroconversion, but this was not validated in larger studies, including retrospective analyses of the MENTOR trial cohort (73, 74). Conversely, a Japanese study showed a high degree of seropositivity for anti-CFH antibodies among patients with MN (up to 77.8% of patients), and that this was associated with renal failure progression but not the degree of proteinuria (75). These discrepant results can be explained by the different cutoff values used to determine positivity for anti-CFH antibodies, with much lower levels recorded in patients with MN than in other disorders classically associated with dysfunction of the alternative pathway of complement, such as atypical hemolytic-uremic syndrome.

Other surface-bound complement regulatory proteins may also be aberrantly expressed in primary MN. As previously described, properdin has been shown to bind directly to proximal tubular epithelial cells in MN patients, amplifying the alternative pathway through stabilization of the alternative pathway C3 convertase C3bBb (57). Furthermore, laser microdissection of glomeruli from kidney biopsies of patients with primary MN consistently showed a reduction in expressed CR1, while MAC expression was increased (41). In the Heymann nephritis model, mice that were deficient in Crry, the murine homologue of MCP, did not develop proteinuria despite formation of subepithelial immune deposits; conversely, injection of anti-Crry antibodies led to rapid induction of heavy proteinuria (26, 76).

Taking these results together, it is clear that dysfunction of complement regulatory proteins of the alternative pathway may contribute to the pathogenesis of MN. One plausible mechanism of renal injury may be antibody-mediated, in a manner akin to that observed in antibody-mediated forms of C3 glomerulopathy. Importantly, such damage may extend beyond glomeruli and may also affect other compartments such as the renal microvasculature or interstitium.

## Utilizing the complement system for non-invasive or adjunctive disease diagnosis

As alluded to above, disease-specific signatures in the pattern of expression of complement activation products are observed in different immune-mediated glomerulonephritides, including MN, FSGS, MCD, IgA nephropathy and lupus nephritis (62). This suggests that, with proper validation, it is plausible that a pattern of complement activation observed in the blood or urine could serve as a liquid biopsy to distinguish a case of suspected MN from other glomerulonephritides. It would be clinically useful to have such diagnostic tools in cases where the kidney biopsy is non-diagnostic due to inadequate sampling or if kidney biopsy is contraindicated for medical reasons. Given the increasing availability of antibody assays such as anti-PLA2R antibody and anti-THSD7A antibody in primary MN, validated complement-based assays could potentially be useful for those cases that are seronegative for all currently known disease-causing antibodies.

Serum C3a and C5a levels are demonstrably higher in patients with MN compared to those with FSGS or MCD (40). However, there is no validated cutoff to distinguish these conditions from one another, and there are also other conditions where complement is activated, such as sepsis. Also, C3a and C5a, and other complement end products are higher in other proliferative glomerulonephritides such as lupus nephritis. This implies that serum C3a or C5a would probably not be sufficiently specific for non-invasive diagnosis.

C4d is an inert complement end-product arising from activation of the classical or mannose-binding lectin pathways, which has most frequently been used to substantiate the diagnosis of antibody-mediated rejection in kidney transplantation. C4d deposits were found in the biopsies of 100% of tested patients with primary MN, but not in any patient with minimal change disease, in a granular pattern around the capillary loops similar to IgG (77). C4d was sufficiently stable to allow for immunofluorescence to be reliably performed on formalin-fixed paraffin-embedded tissue specimens to distinguish MN from other glomerulonephritides, allowing for reliable diagnosis even in cases where there are no viable glomeruli in fresh-frozen sections for conventional immunofluorescence staining for IgG and C3 (78). However, C4d staining may also be strongly positive in other glomerulonephritides with strong complement activation, including lupus nephritis, membranoproliferative glomerulonephritis, and proliferative glomerulonephritis with monoclonal IgG deposits, thus limiting its utility in patients where such conditions are part of the differential diagnosis. Taking the above together, it would appear that current complement-based assays are not specific enough to reliably distinguish MN from other glomerulonephritides. It remains to be seen whether a panel of markers including assays of complement products may have a role to play in disease diagnosis.

## Utilizing the complement system for disease monitoring and prognostication

Given the importance of the complement pathway in the pathogenesis of MN, it follows that monitoring the activity of the complement pathway may have prognostic and treatment implications. For example, C3 levels are typically depressed in the systemic circulation in the context of complement pathway activation; as such, low serum C3 levels have been correlated with poor long-term renal survival in primary MN (79). In multivariate logistic regression analyses, higher immunofluorescence intensity of C3 deposits of human kidney biopsy samples was associated with an increased risk of kidney failure, even after accounting for other factors such as initial glomerular filtration rate (80).

Some serum or urinary markers could be potentially used to predict the subsequent clinical course and identify patients with ongoing immune-mediated kidney damage. For example, urinary C3dg and MAC titres were correlated with “unstable disease course”, defined by the investigators as end-stage renal failure or increase in serum creatinine by 30% to ≥200 umol/L, including patients presenting with heavy proteinuria who received steroid monotherapy (81, 82). The use of urinary MAC excretion alone for prognostication of subsequent renal deterioration was validated in a cohort of patients who received immunosuppressive treatment with steroids and cytotoxic drugs (either chlorambucil or cyclophosphamide) (83). Furthermore, the ratio of urinary MAC to factor H at time of biopsy, before initiation of immunosuppressive treatment, was negatively correlated with creatinine clearance, though not with the level of proteinuria (71). Although proteinuria is typically cited as an important prognostic marker and indication for immunosuppressive treatment in MN, the use of the angiotensin-converting enzyme (ACE) inhibitor captopril controlled proteinuria in MN without affecting urinary excretion of MAC (84). These findings raise doubts as to whether proteinuria control by pharmacological means, in the absence of immunological improvement that is evidenced by reduction of urinary MAC excretion, is a sufficient treatment endpoint in MN. Theoretically, in the patient with MN with residual proteinuria, urinary MAC excretion could be harnessed as a prognostic and therapeutic marker to distinguish between ongoing immune-mediated kidney damage that might benefit from a change of therapy, and proteinuria due to glomerular sclerosis or non-immune-mediated causes such as concomitant diabetic nephropathy.

In using urinary excretion of MAC or other complement activation products to follow the disease activity of MN, an important caveat to note is that such complement activation products may also be falsely elevated in certain conditions. For example, urinary excretion of MAC, as well as activation products such as iC3b and factor Bb, has also been observed in diabetic nephropathy and FSGS with nephrotic-range proteinuria (40, 85). These occur despite the absence of glomerular staining for MAC, raising the possibility that MAC in the urine may be derived from the renal tubules or interstitium rather than from the glomeruli *per se*. In line with this hypothesis, urinary C3d has also been documented in tubulointerstitial nephritis, in the absence of any glomerular pathology (86). MCD may be a rare exception among the glomerulonephritides where urinary excretion of terminal complement complexes is not increased (87). Taken together, these findings suggest that while MAC excretion in the urine may not occur in all glomerular diseases, it is not sufficiently specific for MN. The use of urinary MAC excretion for disease monitoring will have to be validated in MN, and its use limited to select populations with high pre-test probability for active MN, to maximize utility.

## Complement-based therapies for membranous nephropathy

The mainstays of immunomodulatory treatment for primary MN currently comprise calcineurin inhibitors, rituximab, cyclophosphamide, and/or glucocorticoids as per the latest guidelines from the Kidney Disease: Improving Global Outcomes (KDIGO) initiative (88). The choice of therapy depends on the calculated risk of disease non-remission and progression, patient risk, and other real-world considerations such as cost and drug availability. While rituximab or cyclophosphamide are recommended for moderate-to-high risk MN, resistance and non-response to treatment occurs in approximately 30% of patients (89). In some cases this may be related to dosing of anti-CD20 therapy or development of anti-drug antibodies. While strategies such as optimizing dosage of conventional anti-CD20 by using higher doses of rituximab, or using second-generation anti-CD20 agents (such as obinutuzumab and ofatumumab), agents targeted at plasma cells (such as bortezomib or daratumumab), and other B-cell specific agents (such as belimumab) have been proposed, there remains an unmet treatment need for patients who do not respond to conventional therapy (90, 91). Furthermore, reduction in proteinuria often lags behind immunological response in antibody-positive disease. A reduction in anti-PLA2R antibody titre may take weeks after complete B cell depletion by anti-CD20 agents (92). After anti-PLA2R antibodies have decreased or turned negative, proteinuria can then still persist for months (2, 93). Patients may hence still suffer from complications of persistent nephrotic syndrome, and proteinuria itself may have an adverse impact on the renal tubulointerstitium, engendering more chronic tubulointerstitial fibrosis (94). Since new therapies are already highly efficacious in achieving complete B cell depletion, resistance or slow response to treatment would probably be best overcome by identification and therapeutic modulation of other pathophysiological pathways. Although B cell-directed therapies would likely remain the mainstay of treatment in primary MN, therapies targeted at the complement pathway can conceivably be considered in these particular niche scenarios as an adjunctive or rescue therapy, especially if ongoing complement-mediated damage can be demonstrated. Complement-directed therapies are increasingly available and are being trialled in various kidney diseases, including C3 glomerulopathy, IgA nephropathy and others. These could probably be adapted for other complement-mediated kidney diseases including primary MN.

In this regard, the largest trial of the C5 inhibitor eculizumab in MN did not show clinically meaningful improvement with two different dose regimens, although side effects were minimal. This trial has been criticized for using doses far smaller than those used for other complement-mediated diseases, including C3 glomerulopathy or atypical hemolytic-uremic syndrome, and adequate blockade of the complement pathway was demonstrated only in a small fraction of patients (95). Given the significant toxicities of eculizumab in trials of other diseases, including life-threatening infections due to encapsulated organisms, its safety profile must be carefully scrutinized in any future trials in patients with MN.

Use of the small-molecule factor B inhibitor LNP023, also known as iptacopan, in a murine model of MN led to improved proteinuria and halted further disease progression. After the mice were sacrificed, histological analysis showed improvement in glomerulopathy and tubular injury, and absence of C3 staining, confirming successful blockade of the alternative pathway of complement. Importantly, these effects on proteinuria and histology were demonstrated regardless of whether complement blockade was delivered prophylactically prior to induction of MN in mice, or therapeutically after proteinuria had already occurred (96). A recently reported phase II trial of iptacopan in IgA nephropathy showed significant reduction of proteinuria and no cases of severe adverse events or infections due to encapsulated bacteria (97). Another phase II clinical trial comparing LNP023 to rituximab in MN is ongoing (clinical trial code: NCT04154787). Other agents targeting complements or complement regulatory proteins of the alternative pathway include BCX9930 (clinical trial code: NCT05162066), an oral factor D inhibitor.

There have been trials of other complement-based therapies in MN (**Table 1**). APL-2, also known as pegcetacoplan, is a small peptide targeted at C3 and C3b (clinical trial code: NCT03453619) that was trialed for C3 glomerulopathy, IgA nephropathy, primary MN, and lupus nephritis; however, development for the latter two indications was curtailed as the company prioritized development of the drug for C3 glomerulopathy. OMS721, also known as narsoplimab, a monoclonal antibody directed against MASP2 (clinical trial code: NCT02682407), is currently being tested in a phase II clinical trial for MN, and in a phase III trial for IgA nephropathy. While there is growing interest and enthusiasm in targeting the complement systems in MN, one should be mindful of potential adverse effects, such as infection by encapsulated organisms (98). In this regard, in a phase III trial, treatment with avacopan, an oral C5a inhibitor, was not associated with increased risk of infection due to encapsulated organisms including meningococcus in patients with ANCA-associated vasculitis, demonstrating that the potential adverse effects of novel treatments targeting the complement system vary according to the specific role of the complement component targeted (99). Practical considerations in using complement-based therapies include prior vaccination and antimicrobial prophylaxis against meningococcus and pneumococcus, as well as careful application of selection criteria and access (98).

**Table 1:** Complement-based therapies currently under trial for MN.

Name of drug	Target	Phase of development
BCX9930	Factor D	Phase II (NCT05162066)
Eculizumab	C5	Aborted
Iptacopan (LNP023)	Factor B	Phase II (NCT04154787)
Narsoplimab (OMS721)	MASP2	Phase II (NCT02682407)
Pegcetacoplan (APL-2)	C3, C3b	Aborted

## Conclusions

Activation of the complement system is a key contributor to the development of MN. There is a growing understanding of the interplay between disease-causing antibodies, their target antigens, and the complement pathways, in the pathogenesis and clinico-pathological correlations of MN. While there is promising preliminary experience with the use of complements and their activation products in disease monitoring and prognostication, these approaches will have to be validated in well-defined patient cohorts. Therapeutic modulation of the complement system has emerged as a promising treatment strategy in various kidney diseases, and the results in MN are eagerly awaited.

## Author contributions

BS conceptualized the work and drafted the original manuscript. GC, DY, and TC reviewed and edited the manuscript. All authors contributed to the article and approved the submitted version.

## Acknowledgments

DY received research donations from the Wai Im Charitable Foundation and the Chan Sui Kau Family Benefits and Charitable Foundation. DY and TC received research funding support from the Mr and Mrs Tam Wing Fun Edmund Renal Research Fund. TC received research funding support from the Wai Hung Charitable Foundation and Mr S Ho. The funder was not involved in the study design, collection, analysis, interpretation of data, the writing of this article or the decision to submit it for publication.

## Conflict of interest

The authors declare that the research was conducted in the absence of any commercial or financial relationships that could be construed as a potential conflict of interest.

## Publisher’s note

All claims expressed in this article are solely those of the authors and do not necessarily represent those of their affiliated organizations, or those of the publisher, the editors and the reviewers. Any product that may be evaluated in this article, or claim that may be made by its manufacturer, is not guaranteed or endorsed by the publisher.
